# Serum GDF‐15 Levels Correlate With Motor and Nonmotor Symptom Domains and Overall Disease Severity in Patients With Parkinson’s Disease

**DOI:** 10.1155/padi/9949931

**Published:** 2026-04-29

**Authors:** Noriyuki Miyaue, Hayato Yabe, Mina Yasugi, Masahiro Nagai

**Affiliations:** ^1^ Department of Clinical Pharmacology and Therapeutics, Ehime University Graduate School of Medicine, Toon, Ehime, Japan, ehime-u.ac.jp; ^2^ Department of Neurology, Saiseikai Matsuyama Hospital, Matsuyama, Ehime, Japan; ^3^ Department of Rehabilitation, Saiseikai Matsuyama Hospital, Matsuyama, Ehime, Japan

**Keywords:** aging, Growth differentiation factor 15, motor symptoms, nonmotor symptoms, Parkinson’s disease

## Abstract

**Objective:**

Growth differentiation factor 15 (GDF‐15) has emerged as a potential biomarker for neurodegenerative diseases. Although elevated serum GDF‐15 levels have been reported in Parkinson’s disease (PD), their association with clinical features has not been fully characterized.

**Methods:**

We evaluated serum GDF‐15 concentrations in 40 patients with PD and analyzed their relationships with clinical measures, including motor severity (MDS‐UPDRS), quality of life (PDQ‐39), sleep disturbances (PDSS‐2), autonomic symptoms (SCOPA‐AUT), and cognitive function (MoCA‐J).

**Results:**

Higher serum GDF‐15 levels were associated with older age and greater symptom burden across multiple domains. Significant relationships were observed with MDS‐UPDRS Parts I–III and total scores, PDQ‐39 summary index and bodily discomfort index, two different PDSS‐2 domains (motor symptoms at night and PD symptoms at night), and SCOPA‐AUT total and gastrointestinal dysfunction scores. After adjusting for age, the associations between serum GDF‐15 levels and MDS‐UPDRS Part II, Part III, and total scores remained significant. No sex‐related differences were detected. A trend toward lower MoCA‐J scores with increasing GDF‐15 levels was observed but did not reach statistical significance.

**Conclusion:**

Serum GDF‐15 levels are linked to both motor and nonmotor aspects of PD and may reflect overall disease burden. Further longitudinal studies are needed to determine their value for disease monitoring and prognosis.

## 1. Introduction

Parkinson’s disease (PD) is a progressive neurodegenerative disorder characterized by a combination of motor and nonmotor features that substantially affect daily functioning. Although motor manifestations such as bradykinesia, rigidity, and tremor remain central to diagnosis, nonmotor symptoms, including cognitive impairment, sleep disturbances, and autonomic dysfunction, are increasingly recognized as major contributors to reduced quality of life [1]. However, they are often underreported during routine clinical consultations and may remain inadequately managed [2]. Therefore, a comprehensive assessment and targeted management of both motor and nonmotor symptoms are essential for optimizing patient care in PD.

Given the clinical heterogeneity of PD, there is increasing interest in identifying circulating biomarkers that reflect overall disease status. Growth differentiation factor 15 (GDF‐15) has emerged as a candidate molecule in this context. As a stress‐inducible cytokine, GDF‐15 is upregulated in response to mitochondrial dysfunction and cellular injury and is readily measurable in peripheral circulation [3]. Elevated levels have been described across multiple chronic conditions, indicating that GDF‐15 may function as a circulating marker reflecting cumulative systemic and mitochondrial stress [3–7]. In neurodegenerative diseases, including PD, circulating GDF‐15 concentrations have been reported to be increased [8, 9]. However, the clinical implications of this elevation remain incompletely defined. In particular, it is unclear whether GDF‐15 is linked to specific symptom domains or whether it reflects a more global measure of disease burden.

This study sought to clarify the relationship between serum GDF‐15 levels and the clinical manifestations of PD. By evaluating both motor and nonmotor symptom domains, we aimed to clarify the clinical relevance of GDF‐15 as an indicator of disease burden.

## 2. Materials and Methods

### 2.1. Participants

This study reanalyzed individuals diagnosed with PD who were originally enrolled in a prior study examining serum GDF‐15 concentrations across parkinsonian syndromes [10]. PD diagnoses were confirmed according to the Movement Disorder Society (MDS) clinical diagnostic criteria [11]. To minimize confounding factors known to influence circulating GDF‐15 concentrations, individuals were excluded if they had a recent malignancy, active cardiovascular disease, significant renal dysfunction (serum creatinine > 1.5 mg/dL), or chronic liver disease.

The study protocol received approval from the Institutional Review Board for Clinical Research Ethics at Saiseikai Matsuyama Hospital and complied with the Declaration of Helsinki. Written informed consent was obtained from all participants before enrollment.

### 2.2. Clinical Assessment

Demographic and clinical information, including age, sex, disease duration, body weight, body mass index (BMI), and Hoehn and Yahr stage, was recorded. Antiparkinsonian medications were reviewed, and the levodopa equivalent daily dose (LEDD) was calculated using established conversion formulas [12]. Cognitive function was assessed using the Japanese version of the Montreal Cognitive Assessment (MoCA‐J) [13]. All participants were assessed during the ON medication state using the MDS revision of the Unified Parkinson Disease Rating Scale (MDS‐UPDRS), which includes Part I (nonmotor aspects of experiences of daily living), Part II (motor aspects of experiences of daily living), Part III (motor examination), and Part IV (motor complications) [14]. Quality of life was evaluated using the PD Questionnaire‐39 (PDQ‐39), which consists of a summary index (SI) and eight domain indices: mobility, activities of daily living, emotional well‐being, stigma, social support, cognition, communication, and bodily discomfort [15]. Sleep‐related disturbances were evaluated using the PD Sleep Scale‐2 (PDSS‐2), which includes three domains: motor symptoms at night, PD symptoms at night, and disturbed sleep [16]. Autonomic symptoms were assessed using the Scale for Outcomes in PD for Autonomic (SCOPA‐AUT) symptoms, which consists of six domains: gastrointestinal dysfunction, urinary dysfunction, cardiovascular dysfunction, thermoregulatory dysfunction, pupillomotor dysfunction, and sexual dysfunction [17].

### 2.3. Measurement of Serum GDF‐15

Blood samples were collected and processed under standardized conditions. After centrifugation, serum was separated and stored at −80°C until analysis. GDF‐15 concentrations were determined using a commercially available enzyme‐linked immunosorbent assay (ELISA) kit according to the manufacturer’s instructions (R&D Systems, Minneapolis, MN, USA). Each sample was analyzed in duplicate, and the mean value was used for subsequent analyses.

### 2.4. Statistical Analysis

Continuous variables are presented as mean ± standard deviation. Between‐group comparisons for continuous variables were conducted using the Mann–Whitney *U* test, and the chi‐square test was applied for categorical variables. Associations between serum GDF‐15 levels and clinical parameters were examined using Spearman’s rank correlation. Multivariable regression analyses were then performed, incorporating age, sex, and disease duration as potential covariates; only variables demonstrating significant univariate associations with GDF‐15 were retained in the final models. Statistical significance was defined as a two‐sided *p* value < 0.05. All analyses were performed using R software (Version 4.4.0; The R Foundation for Statistical Computing, Vienna, Austria).

## 3. Results

A total of 40 patients with PD were included in this study (17 females and 23 males). The mean age was 72.62 ± 8.58 years, the mean disease duration was 6.03 ± 4.47 years, and the mean Hoehn and Yahr stage was 2.95 ± 0.78. The mean serum GDF‐15 level was 1372.33 ± 542.70 pg/mL. The detailed clinical characteristics of all patients are presented in Table 1.

**TABLE 1:** Clinical characteristics of the patients.

	**PD (*n* = 40)**

Age (years)	72.62 (8.58)
Female/male	17/23
Disease duration (years)	6.03 (4.47)
Hoehn and Yahr stage	2.95 (0.78)
Body weight (kg)	56.67 (10.00)
Body mass index (kg/m^2^)	22.07 (3.05)
Levodopa equivalent daily dose (mg/day)	619.32 (320.26)
Antiparkinsonian medication, *n* (%)	
Levodopa	40 (100.0)
Dopamine agonist	21 (52.5)
COMT inhibitor	9 (22.5)
MAO‐B inhibitor	17 (42.5)
Amantadine	11 (27.5)
Istradefylline	1 (2.5)
Zonisamide	20 (50.0)
Trihexyphenidyl	5 (12.5)
MoCA‐J score	24.08 (4.12)
Serum GDF‐15 level (pg/mL)	1372.33 (542.70)

*Note:* Values are presented as mean (standard deviation) or *n* (%). MAO‐B, Monoamine oxidase‐B; MoCA‐J, Japanese version of the Montreal Cognitive Assessment.

Abbreviations: COMT, catechol‐O‐methyl transferase; GDF‐15, Growth differentiation factor 15; PD, Parkinson’s disease.

We investigated the association between serum GDF‐15 levels and clinical parameters, as well as motor and nonmotor symptom scores (Table 2). Serum GDF‐15 levels were significantly positively correlated with age (*r* = 0.375; *p* = 0.017) but not with disease duration, BMI, or LEDD. In addition, serum GDF‐15 levels showed a trend toward a negative correlation with cognitive performance as assessed by MoCA‐J scores (*r* = −0.252), although this association did not reach statistical significance (*p* = 0.117). In MDS‐UPDRS, serum GDF‐15 levels were significantly correlated with Part I (*r* = 0.318; *p* = 0.045), Part II (*r* = 0.403; *p* = 0.010), and Part III scores (*r* = 0.495; *p* = 0.001), as well as total scores (*r* = 0.493; *p* = 0.001) (Figure 1(a)). Furthermore, serum GDF‐15 levels were significantly correlated with PDQ‐39 SI scores (*r* = 0.335; *p* = 0.035) and bodily discomfort index scores in PDQ‐39 (*r* = 0.467; *p* = 0.002) (Figure 1(b)). Regarding PDSS‐2, while total scores were not significantly correlated with serum GDF‐15 levels (*r* = 0.246; *p* = 0.126), significant correlations were observed for motor symptoms at night and PD symptoms at night scores (*p* = 0.041 and *p* = 0.044, respectively). In SCOPA‐AUT, serum GDF‐15 levels were significantly correlated with total scores (*r* = 0.395; *p* = 0.012) and gastrointestinal dysfunction scores (*r* = 0.491; *p* = 0.001) (Figure 1(c)).

**TABLE 2:** Results of correlation analysis with serum GDF‐15 levels.

	***r***	**p**

Age	0.375	0.017
Body weight	0.060	0.714
BMI	0.005	0.976
Disease duration	0.292	0.067
LEDD	0.245	0.128
MoCA‐J score	−0.252	0.117
MDS‐UPDRS Part I score	0.318	0.045
MDS‐UPDRS Part II score	0.403	0.010
MDS‐UPDRS Part III score	0.495	0.001
MDS‐UPDRS Part IV score	−0.016	0.923
MDS‐UPDRS part total score	0.493	0.001
PDQ‐39 SI score	0.335	0.035
Mobility index score	0.299	0.061
Activities of daily living index score	0.235	0.145
Emotional well‐being index score	0.078	0.634
Stigma index score	0.131	0.421
Social support index score	0.084	0.606
Cognition index score	0.288	0.071
Communication index score	0.239	0.137
Bodily discomfort index score	0.467	0.002
PDSS‐2 total score	0.246	0.126
Motor symptoms at night score	0.325	0.041
PD symptoms at night score	0.321	0.044
Disturbed sleep score	0.061	0.707
SCOPA‐AUT total score	0.395	0.012
Gastrointestinal dysfunction score	0.491	0.001
Urinary dysfunction score	0.105	0.519
Cardiovascular dysfunction score	0.227	0.160
Thermoregulatory dysfunction score	0.113	0.488
Pupillomotor dysfunction score	0.116	0.476
Sexual dysfunction score	−0.042	0.798

*Note:* LEDD, levodopa equivalent dose; MDS‐UPDRS, Movement Disorder Society–sponsored revision of the Unified Parkinson Disease Rating Scale; MoCA‐J, Japanese version of the Montreal Cognitive Assessment; SCOPA‐AUT, Scale for Outcomes in Parkinson’s disease for Autonomic symptoms.

Abbreviations: BMI, body mass index; GDF‐15, Growth differentiation factor 15; PDQ‐39 SI, Parkinson’s Disease Questionnaire‐39 summary index; PDSS‐2, Parkinson’s Disease Sleep Scale‐2; SE, standard error.

FIGURE 1Correlation with serum GDF‐15 levels. Serum GDF‐15 levels were significantly correlated with MDS‐UPDRS total score (*r* = 0.493; *p* = 0.001) (a), PDQ‐39 SI score (*r* = 0.335; *p* = 0.035) (b), and SCOPA‐AUT total score (*r* = 0.395; *p* = 0.012) (c). GDF‐15: Growth differentiation factor 15; MDS‐UPDRS: Movement Disorder Society–sponsored revision of the Unified Parkinson Disease Rating Scale; PDQ‐39 SI: Parkinson’s Disease Questionnaire‐39 summary index; SCOPA‐AUT: Scale for Outcomes in Parkinson’s disease for Autonomic symptoms.

**Figure:**
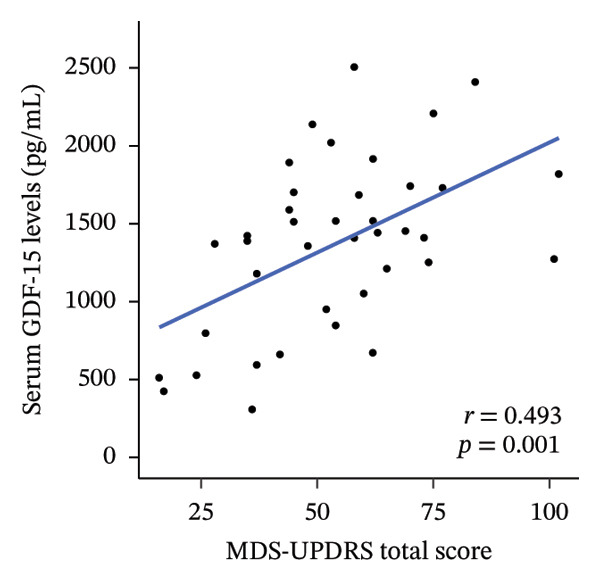
(a)

**Figure:**
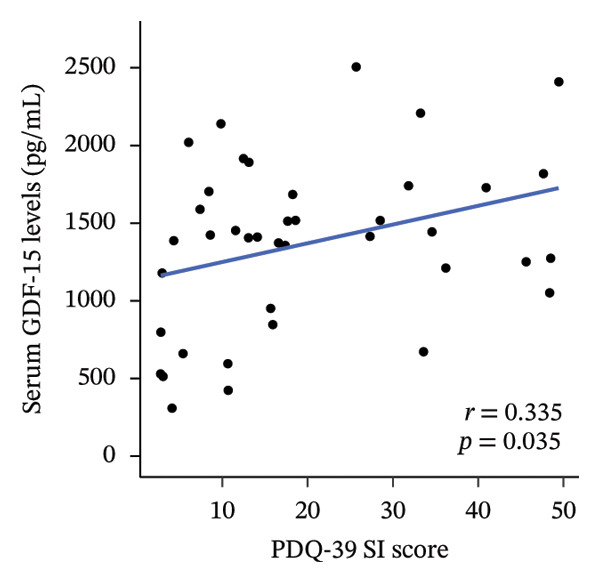
(b)

**Figure:**
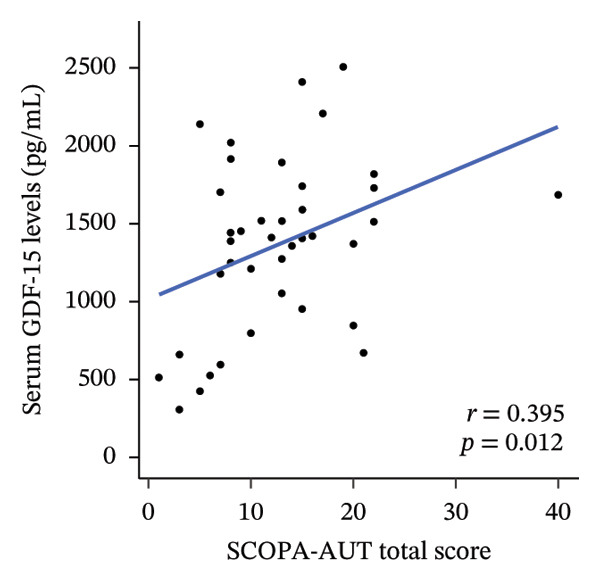
(c)

Next, we compared clinical characteristics, motor and nonmotor symptoms, and serum GDF‐15 levels between female and male patients (Supporting Table 1). No significant difference was observed in age between female (74.41 ± 9.32 years) and male patients (71.30 ± 7.95 years) (*p* = 0.167). Female patients had significantly lower body weight than male patients (*p* < 0.001), while no significant difference was observed in BMI (*p* = 0.122). Male patients exhibited significantly higher MDS‐UPDRS Part II scores (*p* = 0.021), communication index scores in PDQ‐39 (*p* = 0.045), and sexual dysfunction in SCOPA‐AUT (*p* = 0.043) compared to female patients. Serum GDF‐15 levels did not significantly differ between female (1285.82 ± 469.27 pg/mL) and male patients (1436.26 ± 593.22 pg/mL) (*p* = 0.318).

Multivariate analysis demonstrated that age, but not sex or disease duration, was independently significantly associated with serum GDF‐15 levels (*p* < 0.001) (Table 3). Based on this result, we conducted a multivariate analysis using age as the explanatory variable and serum GDF‐15 levels as the dependent variable. The analysis revealed that serum GDF‐15 levels were significantly associated with LEDD (*p* = 0.031), MDS‐UPDRS Part II (*p* = 0.042), Part III (*p* = 0.007), and total scores (*p* = 0.008).

**TABLE 3:** Multivariate analysis of serum GDF‐15 levels and clinical characteristics.

	**Estimate**	**Standard error**	***t* value**	**p**

Intercept	−1237.977	679.602	−1.822	0.077
Age	32.466	8.923	3.638	< 0.001
Sex = male	189.349	159.545	1.187	0.243
Disease duration	23.827	17.594	1.354	0.184

Abbreviation: GDF‐15, Growth differentiation factor 15.

## 4. Discussion

In this study, we observed that serum GDF‐15 levels increased with age in patients with PD and were positively associated with multiple clinical features, including impaired quality of life, more severe motor symptoms, sleep‐related difficulties, and autonomic dysfunction. Notably, the associations with MDS‐UPDRS Part II, Part III, and total scores remained significant after adjustment for age, indicating that GDF‐15 is not merely a surrogate marker of chronological aging but may capture disease‐related burden.

Although GDF‐15 is widely recognized as an age‐associated biomarker [18, 19], its elevation in PD may also reflect underlying pathological processes. Mitochondrial dysfunction and cellular stress responses, both central features of PD, are known to induce GDF‐15 expression [20]. Therefore, increased circulating levels may represent the cumulative impact of these processes rather than a disease‐specific mechanism.

Although sex‐related differences in circulating GDF‐15 levels have been reported in non‐PD populations [21], we did not observe such differences in this cohort, consistent with previous findings in PD [8, 22]. This discrepancy may reflect disease‐specific factors or limited statistical power and warrants further investigation.

Our analysis further demonstrated that serum GDF‐15 levels were related to both subjectively reported and objectively evaluated motor impairments, as reflected by MDS‐UPDRS Parts II and III scores. Prior studies investigating the relationship between serum GDF‐15 levels and motor symptoms, as assessed by UPDRS Part III score, have yielded inconsistent results [8, 23]. The present findings suggest that GDF‐15 may capture multiple aspects of motor dysfunction, particularly after accounting for age; however, given the multiple correlation analyses performed, these results should be regarded as exploratory rather than definitive.

With respect to nonmotor symptoms, only one prior investigation evaluated their relationship to GDF‐15 and found associations with depressive symptoms, sexual dysfunction, and excessive daytime sleepiness [22]. In contrast, this study demonstrated that elevated serum GDF‐15 levels were significantly associated with increased severity of nocturnal motor and PD‐related symptoms, autonomic dysfunction, particularly gastrointestinal disturbances, as well as reduced quality of life. Among these domains, autonomic dysfunction showed the most consistent association with serum GDF‐15 levels. It should be noted, however, that correlations observed with PDQ‐39 and sleep‐related measures may, at least in part, reflect indirect effects of motor symptom severity rather than nonmotor symptom burden. Therefore, these results should be interpreted with caution. Future studies incorporating comprehensive nonmotor assessment tools, such as the Non‐Motor Symptoms Scale, may help to better disentangle these relationships.

The relationship between GDF‐15 and cognitive function remains uncertain [24, 25]. Although higher GDF‐15 levels tended to be associated with lower cognitive scores, the lack of statistical significance suggests that circulating GDF‐15 may not be a sensitive marker of cognitive impairment in PD. These results suggest that circulating GDF‐15 may have limited utility as a marker of cognitive impairment in PD.

Interestingly, while GDF‐15 did not correlate with LEDD in univariate analysis, LEDD became a significant predictor after controlling for age. This may be influenced by the fact that in elderly patients, the increase in LEDD with disease progression is less pronounced compared to younger patients [26]. These findings may indicate that higher GDF‐15 levels are associated with greater clinical severity and may reflect an association with increased dopaminergic treatment requirements.

The biological role of GDF‐15 in PD remains to be established. While experimental evidence has suggested potential neuroprotective effects under certain conditions, other studies have not demonstrated a direct impact on dopaminergic neuron survival [27, 28]. Taken together, these findings support the interpretation that GDF‐15 primarily functions as a marker of systemic stress rather than a mediator of neurodegeneration.

This study has several limitations. First, the relatively small sample size and single‐center design may limit statistical robustness, particularly in the context of multiple correlation analyses. Second, the absence of a healthy control group required reliance on comparisons with previously published data. Third, due to the cross‐sectional design, it remains unclear whether serum GDF‐15 reflects disease progression or predicts clinical outcomes such as mortality or institutionalization. Finally, GDF‐15 is not disease‐specific and is elevated in several neurodegenerative disorders. Accordingly, serum GDF‐15 should be interpreted as a complementary marker reflecting disease burden rather than a diagnostic biomarker for PD. In addition, most participants had moderate‐stage PD, limiting the generalizability of findings to earlier disease stages. Because elevated GDF‐15 has been associated with increased mortality risk in large cohorts [29, 30], future longitudinal studies should investigate whether GDF‐15 predicts clinical progression or prognosis in PD.

## 5. Conclusions

Serum GDF‐15 levels were associated with several clinical features of PD, including reduced quality of life, autonomic dysfunction, and nocturnal symptoms. Although GDF‐15 is strongly influenced by aging, our findings suggest that it may also reflect PD‐related disease burden. However, given its lack of disease specificity and the cross‐sectional nature of this study, the direct clinical applicability of serum GDF‐15 measurement remains limited at present. Larger, longitudinal studies, particularly those including prodromal and early‐stage parkinsonian disorders, are warranted to determine whether GDF‐15 may have utility as a complementary biomarker for disease monitoring or prognosis in PD.

## Funding

This research was supported by JSPS KAKENHI (Grant no. JP21K15699).

## Conflicts of Interest

The authors declare no conflicts of interest.

## Supporting Information

Supporting Table 1. Clinical characteristics, serum GDF‐15 levels, and motor/nonmotor symptoms compared between female and male patients.

## Supporting information


**Supporting Information** Additional supporting information can be found online in the Supporting Information section.

## Data Availability

The data that support the findings of this study are available on request from the corresponding author. The data are not publicly available due to privacy or ethical restrictions.
