# Supplementary material for: Serum GDF‐15 Levels Correlate With Motor and Nonmotor Symptom Domains and Overall Disease Severity in Patients With Parkinson’s Disease
Source: Parkinsons Dis. 2026 Apr 29;2026:9949931. doi: 10.1155/padi/9949931 (PMC13129218; doi:10.1155/padi/9949931)
Supplement: Supplementary file 1 — Supporting Information Additional supporting information can be found online in the Supporting Information section. [file PADI-2026-9949931-s001.docx]

**Supplementary Table 1. Clinical characteristics, serum GDF-15 levels, and motor/non-motor symptoms compared between female and male patients**

|  | **Female** | **Male** | *p* |
| --- | --- | --- | --- |
| n | 17 | 23 |  |
| Age, years | 74.41 (9.32) | 71.30 (7.95) | 0.167 |
| Body weight, kg | 49.15 (8.81) | 62.23 (6.69) | <0.001 |
| BMI, kg/m^2^ | 21.31 (3.73) | 22.63 (2.36) | 0.122 |
| Disease duration, years | 4.53 (2.58) | 7.13 (5.26) | 0.110 |
| LEDD, mg/day | 588.62 (210.40) | 642.00 (385.17) | 0.827 |
| MoCA-J score | 24.65 (4.29) | 23.65 (4.03) | 0.323 |
| Serum GDF-15 level, pg/mL | 1285.82 (469.27) | 1436.26 (593.22) | 0.318 |
| MDS-UPDRS part I score | 8.76 (3.53) | 10.91 (5.52) | 0.197 |
| MDS-UPDRS part II score | 8.65 (5.80) | 14.39 (7.79) | 0.021 |
| MDS-UPDRS part III score | 27.24 (10.91) | 33.13 (8.95) | 0.100 |
| MDS-UPDRS part IV score | 1.47 (1.84) | 1.09 (2.04) | 0.319 |
| MDS-UPDRS part total score | 46.12 (17.36) | 59.52 (20.13) | 0.065 |
| PDQ-39 SI | 16.93 (13.47) | 22.35 (15.68) | 0.239 |
| Mobility index | 24.56 (25.58) | 34.67 (25.42) | 0.193 |
| Activities of daily living index | 15.44 (20.19) | 29.53 (28.20) | 0.057 |
| Emotional well-being index | 13.24 (9.55) | 16.74 (14.53) | 0.630 |
| Stigma index | 12.87 (17.18) | 18.21 (16.95) | 0.194 |
| Social support index | 3.92 (8.90) | 7.61 (15.27) | 0.500 |
| Cognition index | 27.21 (19.63) | 29.62 (18.39) | 0.500 |
| Communication index | 6.86 (10.31) | 20.29 (21.44) | 0.045 |
| Bodily discomfort index | 31.37 (23.85) | 22.10 (18.57) | 0.203 |
| PDSS-2 total score | 11.35 (10.04) | 13.52 (7.51) | 0.087 |
| Motor symptoms at night | 4.82 (3.21) | 6.00 (3.16) | 0.151 |
| PD symptoms at night | 1.47 (3.28) | 1.61 (1.92) | 0.076 |
| Disturbed sleep | 5.06 (4.13) | 5.91 (3.70) | 0.333 |
| SCOPA-AUT total score | 11.47 (5.47) | 13.78 (8.22) | 0.476 |
| Gastrointestinal dysfunction | 4.12 (3.35) | 5.17 (5.71) | 0.640 |
| Urinary dysfunction | 5.24 (3.53) | 5.61 (3.24) | 0.657 |
| Cardiovascular dysfunction | 0.82 (1.01) | 1.26 (1.32) | 0.265 |
| Thermoregulatory dysfunction | 1.06 (1.30) | 0.70 (0.88) | 0.486 |
| Pupillomotor dysfunction | 0.24 (0.44) | 0.26 (0.54) | 0.955 |
| Sexual dysfunction | 0.00 (0.00) | 0.78 (1.86) | 0.043 |

BMI, body mass index; GDF-15, growth differentiation factor 15; LEDD, levodopa equivalent dose; MDS-UPDRS, Movement Disorder Society-sponsored revision of the Unified Parkinson Disease Rating Scale; MoCA-J, Montreal Cognitive Assessment; PDQ-39 SI, Parkinson's Disease Questionnaire-39 summary index; PDSS-2, Parkinson's Disease Sleep Scale-2; SCOPA-AUT, Scale for Outcomes in Parkinson’s disease for Autonomic symptoms; SE, standard error.
